# Systematic review of depression in mild traumatic brain injury: study protocol

**DOI:** 10.1186/s13643-016-0196-6

**Published:** 2016-02-06

**Authors:** Chris Lepage, Tina Yuan, Stephanie Leon, Shawn Marshall, Patrick Labelle, Mark Ferland

**Affiliations:** School of Psychology, University of Ottawa, Ottawa, ON Canada; The Children’s Hospital of Eastern Ontario Research Institute, Ottawa, ON Canada; The Ottawa Hospital Research Institute, Ottawa, ON Canada; The Robin Easey Centre, Ottawa Hospital Rehabilitation Centre, Ottawa, ON Canada

**Keywords:** Depression, Mild traumatic brain injury, Outcomes, Prognosis, Systematic review

## Abstract

**Background:**

Of the over 1 million reported cases of traumatic brain injuries reported annually in the USA, a sizeable proportion are characterized as mild. Although it is generally well-accepted that most people who suffer a mild traumatic brain injury recover within 1 to 3 months, a proportion of individuals continue to experience physiological, psychological, and emotional symptoms beyond the expected window of recovery. Depression is commonly reported following mild traumatic brain injury; however, its course, consequences, and prognostic factors remain to be well understood.

**Methods:**

A systematic review will be conducted of available prospective longitudinal studies of adult mild traumatic brain injury-related depression. The aim of the systematic review is to describe the course of mild traumatic brain injury-related depression, along with its prognostic factors and health consequences. The review will comply with the Preferred Reporting Items for Systematic Reviews and Meta-analyses guidelines. A thorough database search of peer-reviewed publications in English and French will be conducted in PubMed, Medical Literature Analysis and Retrieval System Online (MEDLINE), PsycINFO, Cumulative Index to Nursing and Allied Health Literature (CINAHL), Cochrane, Embase, Scopus, Erudit, and Cairn. Independent investigators will perform study selection and data extraction. Risk of bias will be assessed using the Quality in Prognosis Studies tool, and methodological quality will be evaluated using a system inspired by the Scottish Intercollegiate Guidelines Network Methodology. Results will be presented through qualitative description and tabulation.

**Discussion:**

This will be the first systematic review conducted with the aim of describing the course, prognostic factors, and health-related outcomes of depression in adults who have suffered a mild traumatic brain injury. The findings of the planned systematic review have the potential to guide research and clinical practice to effectively develop and implement evidence-based interventions aimed at preventing and alleviating mild traumatic brain injury-related depression.

**Systematic review registration:**

PROSPERO CRD42015019214

## Introduction

Of the estimated 1.4 million new cases of traumatic brain injury in the USA per year, approximately 85 % are considered mild [1]. The annual costs of brain injury—including treatment, rehabilitation, and lost productivity—are tremendous [2]. Although there is no universally accepted definition of mild traumatic brain injury (mTBI), it is usually characterized by a traumatic injury to the head with at least one of the following: loss of consciousness for less than 30 min, limited memory impairment, altered mental state (e.g., confusion), or neurological dysfunction (e.g., focal signs, seizure) [3]. Most patients who suffer an mTBI are expected to recover within 3 months [4]; however, a subset of patients experience symptoms for longer periods. These symptoms are heterogeneous and include physical, cognitive, and affective disturbances, many of which overlap with those that characterize depression. Depression is commonly reported in patients presenting at emergency rooms [5] and post-injury referral services [6] for mTBI; however, the course and prognosis of depression following mTBI appears to vary widely [7]. Furthermore, little is known about the influence that depression has on health-related outcomes and the rate of recovery from mTBI.

Reviews of interventions for depression have been conducted systematically and narratively in traumatic brain injury (TBI) and mTBI, respectively; yet, the course of depression as it relates to mTBI has not been systematically reviewed. Much remains to be uncovered about the clinical, behavioral, and physiological factors that are associated with the manifestation of depression after an mTBI. Thus, we will synthesize prospective longitudinal studies of mTBI-related depression to describe its course, ascertain its prognostic factors, and describe its health consequences.

## Methods

### Eligibility criteria

#### Inclusionary criteria

To be included in the systematic review, studies must have been peer-reviewed, must be published in English or French, and must have investigated depression in adult patients (>18 years of age) reported to have sustained an mTBI. Dissertations along with unpublished studies and data will be eligible. All diagnostic criteria used to identify mTBI will be evaluated. All definitions of depression will be considered, including those of the various iterations of the Diagnostic and Statistical Manual of Mental Disorders [8] and International Classification of Disease [9]. Similarly, all measures of depression will be accepted, including single or multiple item depression scales, and the presence or absence of depression determined via interview. All intervention and effectiveness studies of longitudinal design, observational cohort, and case control-design studies will be considered for review.

#### Exclusionary criteria

Studies investigating penetrating brain injuries, birth injuries, brain damage resulting from stroke or other cerebrovascular accidents, shaken baby syndrome, or moderate-to-severe closed-head injuries will be excluded. Similarly, the following will be excluded from this study: cross-sectional studies, case reports, pediatric studies, non-systematic review papers (e.g., narrative reviews), clinical review papers, letters to the editor and editorials without data, studies using nonhuman subjects, and articles with no primary data. Reference lists of review papers will be searched to ensure all relevant literature will be included.

### Information sources and search strategy

A robust search strategy to examine depression in mTBI was developed in collaboration with disease experts and an information specialist (PL). We will search the following electronic bibliographic databases since their inception for peer-reviewed studies: PubMed, Medical Literature Analysis and Retrieval System Online (MEDLINE), PsycINFO, Cumulative Index to Nursing and Allied Health Literature (CINAHL), Cochrane, Embase, Scopus, Erudit, and CAIRN. The search strategy will include the terms mild traumatic brain injury, traumatic brain injury, mTBI, TBI, concussion, major depression, and dysthymia, as keywords or subject headings. Only publications in English and French will be sought. An example of a search strategy for this review is shown in Table 1.

**Table 1:** Example of search strategy—MEDLINE

Term number	Search terms	Results
1	exp brain injuries/	
2	craniocerebral trauma/	
3	exp head injuries, closed/	
4	exp skull fractures/	
5	(mild traumatic brain injur^a^ or traumatic brain injur^a^ or mtbi or tbi).ti,ab	
6	(concuss^a^ or postconcuss^a^ or post-concuss^a^).ti,ab	
7	(mild head injur^a^ or minor head injur^a^ or minor brain injur^a^).ti,ab	
8	or/1-7	95,069
9	depression/	
10	exp depressive disorder/	
11	depressi^a^.ti,ab	
12	dysthym^a^.ti,ab	
13	or/9-12	313,682
14	and/8,13	2182
15	exp animals/ not humans/	
16	14 not 15	1982
17	limit 16 to (english or french)	1819

^a^used to denote wildcard

### Study records

#### Data management

Search results, including abstracts and full-text articles, will be managed via EndNote and Paperpile. Apache OpenOffice Calc will be used to store abstracted data.

#### Selection process

Study selection will be conducted in a two-stage process. First, two evaluators (CL and TY) will independently, and in duplicate, screen study titles and abstracts of the obtained citations for potential inclusion. Each reviewer will record his decision in an Excel file. Disagreements about the articles to include will be resolved by a consensus between the reviewers. When consensus is not possible, the full article will be obtained, and a subsequent attempt for agreement will be made. If no consensus is achieved, a third review will resolve the disagreement. Next, studies identified as potentially relevant will undergo full-text screening by both evaluators. A third evaluator will resolve disagreements regarding study eligibility following full-text review.

#### Data collection process

Two reviewers (CL and TY) will independently, and in duplicate, extract data from studies that meet the selection criteria. A third evaluator will resolve data discrepancies between the two evaluators. Data will be entered into collection forms grouped by study design.

Where data are missing, primary study authors will be contacted. In the case of unavailable data, the proportion of missing data will be reported when possible, as well as possible reasons. In the cases of duplicate publications and multi-paper studies of the same sample, all available data will be examined contemporaneously.

#### Data items

Data from observational studies will be used to achieve the primary aims of this systematic review (i.e., describe the course, predictors, and health consequences of mTBI-related depression). In the case of intervention studies, variables related to depression and mTBI will be abstracted only from untreated and no-effect groups. The following data will be abstracted to summarize study-specific details and to address the aims of the review:

1. Study characteristics (i.e., authors names, publication year, country, setting, design, sample size, sample referral source, depression measure used, definition of mTBI, number of participants assessed at each time-point, time between assessments, and time from injury to follow-up)
2. Participant characteristics (i.e., mean age, sex, history of pre-mTBI depression, localization of injury, injury severity, and mechanism of injury)
3. Medications used by or administered to participants
4. Results (i.e., incidence, prevalence, magnitude of depression, comorbidities, and other factors, such as prognostic factors and health-related outcomes)

### Outcomes and prioritization

The goals of this systematic review are to (1) synthesize evidence on the clinical course of mTBI-related depression, (2) describe the factors that impact depression for individuals with mTBI, and (3) detail the consequences of depression in mTBI. To evaluate the clinical course of depression, change in the frequency of depression across time-points will be reported. Prognostic factors will comprise those aspects of mTBI or individual-level characteristics statistically associated (e.g., via relative risk, odds ratio) with depression. Data will be extracted and reported for every follow-up time-point where the hypothesized predictor variable was assessed within each cohort. To report on the health outcomes of mTBI-related depression, the negative effects associated with depression after mTBI (i.e., quality of life, mortality, costs, etc.) at each time-point will be evaluated in the same way as prognostic factors.

### Risk of bias in individual studies

Two reviewers will independently assess risk of bias in primary studies using the Quality in Prognosis Studies (QUIPS) tool [10, 11]. Each study will be assessed on the six possible sources of bias outlined by the QUIPS tool: study participation, attrition, confounding, analysis, reporting, outcome measurement, and prognostic factor measurement. Sampling method will be examined as an additional potential source of bias. Biased sampling methods will include the use of recruitment signs, snowball sampling, overzealous inclusion/exclusion criteria, self-selection, and unmentioned sampling methods. Recruitment source (e.g., emergency department, psychologist’s office) will be evaluated for bias, as depression may be systematically overrepresented in some sites over others.

Potential sources of bias will be rated as either “yes,” “partly,” “no,” or “unsure.” Each study will then be given an overall bias rating of “high,” “moderate,” or “low.” Studies with an overall bias of high or moderate will be excluded from the systematic review due to an unacceptable level of bias when sensitivity analyses suggest that they may compromise the conclusions of the review [12]. A supplementary table will be provided that will detail the reasons for study exclusion.

### Methodological quality in individual studies

Methodological quality will be evaluated using a system inspired by the Scottish Intercollegiate Guidelines Network Methodology (www.sign.ac.uk) and as implemented by Mollayeva et al. [13]. With respect to the quality criteria proposed by Hayden et al. [10], high-quality studies (+++) will be those that meet all criteria or, at most, have one partially scored criterion. Studies will be rated as good quality (++) when the majority of the criteria are met. When little criteria are met, but at least one rating of “yes” was obtained, studies will be considered fair quality (+). The results of the quality assessment will be reported in a table format. Additional methodological quality criteria will be applied pertinent to the aims of our systematic review. Specifically, studies will be rated along the following dimensions:

1. History of pre-mTBI depression: Studies looking at samples that were negative for pre-mTBI depression will be rated as having “met the criteria.” Next, studies that controlled for pre-existing depression will be rated as “partially” meeting these criteria. Finally, studies that did not control for pre-existing depression or did not report on pre-mTBI depression will be rated as “not meeting the criteria.”
2. Time since injury: Studies reporting time since injury and having a homogeneous sample will be rated as having “met the criteria.” Next, studies with heterogeneous samples but that controlled for time since injury will be rated as “partially meeting criteria.” Finally, studies that did not take time-since-injury into account will be rated as “not meeting criteria.”
3. Presence of a valid comparison group: Studies that included a comparison group that had the same base risk and was drawn from the same population will be rated as having “met the criteria.” Next, studies that included a comparison group that was not drawn from the same population will be rated as “partially” meeting criteria. Finally, studies without a comparison group (e.g., case studies) will be rated as “not meeting criteria.”
4. Use of a valid, reliable, and responsive measure of mTBI: Studies with a clear definition of mTBI and the same measures across participants (e.g., physician rated Glasgow Coma Scale) will be rated as having “met the criteria.” Studies with a poor or vague definition of mTBI and/or omission of a valid measure (i.e., self-report) will be rated as “not meeting the criteria”.
5. Use of valid, reliable, and responsive measure of depression: Studies that included a clear definition of depression and used the same measure of depression across participants will be rated as having “met the criteria.” Studies with a poor or vague definition of depression and/or an invalid measure (i.e., any proxy measure) will be rated as “not meeting the criteria.”

### Data

#### Synthesis

Studies will first be qualified according to our inclusion and exclusion criteria, followed by an analysis of risk of bias. The abstracted data from the selected studies will be synthesized and presented according to the primary aims of the review. Synthesis by means of tabulation and qualitative description of the results from studies with at least fair quality will be provided; however, studies failing to meet our methodological quality criteria will be only briefly summarized.

The methods used to diagnose depression will be reported (e.g., single measure, clinical interview). The definitional criteria of mTBI will also be provided (e.g., World Health Organization (WHO) guidelines, loss of consciousness under 30 min, post-traumatic amnesia lasting less than 24 h, confusional state). Similarly, diagnostic methods used to ascertain history of mTBI will be reported (e.g., self-report, medical records).

##### Course of depression

The overall percentage, range, and median will be calculated for the studies reporting on depression incidence. Depression status will be identified and reported as “increased” depression, “reduced” depression or “no change.”

##### Prognostic factors

Significant prognostic factors will be summarized by having any association statistic (e.g., correlation, odds ratio, relative risk) reported along with related significance values. Point estimates for each prognostic factor will be reported, taking into consideration power through differential weighting of studies based on sample size. Results will be reported for every time-point where the prognostic factor was assessed within each cohort. Factors that will be explored but found to be unrelated to depression in mTBI will be reported.

##### Health-related outcomes

The causal relationship between mTBI-related depression and health outcomes may be difficult to establish; thus, the following criteria will be reported: (1) health outcome response to changes in depression over time; (2) absence of alternative origins, where available; and (3) temporal associations. Health-related outcomes that have been associated to mTBI-related depression will be determined.

Confounding factors with the potential to impact the generalizability of the study and the interpretation of the results will be detailed. We anticipate—due to the heterogeneity in the definition and assessment of depression in the mTBI literature—a meta-analysis will not be feasible; however, a final determination will be made once all study data have been extracted.

## Discussion

To our knowledge, this is the first systematic review with the aim of describing the clinical course, prognostic factors, and health-related outcomes of depression in adults who have suffered an mTBI. The findings of this systematic review have the potential to impact practice, future research, and policy. The results from this review may be used to guide research and clinical practice to most effectively develop and implement evidence-based interventions aimed at preventing and alleviating mTBI-related depression. The strengths of the study include a comprehensive literature search, the use of measures to protect against bias in study selection, and rigorous evaluation of methodological quality.

There are some limitations to the planned systematic review. First, only studies written in English and French will be used in this review; thus, relevant studies in other languages will be excluded. Assessing course, prognosis, and health outcomes in mTBI-related depression will be a challenge because of the breadth of studies. Potential sources of bias will include participant selection and recruitment, which will hamper the ability to determine incidence of depression [14]. Similarly, lack of control for confounding variables in many studies will make it difficult to ascertain the actual variability explained by prognostic factors [14].

Given the prevalence of mTBI, it will be important for the findings of this study to be widely disseminated in relevant healthcare, scientific, and medical fields. As such, the findings from this systematic review will be shared via scientific peer-reviewed journals, conference presentations, proceedings, and other relevant meetings.

## Abbreviations

mTBI: mild traumatic brain injury
TBI: traumatic brain injury
QUIPS: Quality in Prognosis Studies
WHO: World Health Organization
